# *Blautia* genus associated with visceral fat accumulation in adults 20–76 years of age

**DOI:** 10.1038/s41522-019-0101-x

**Published:** 2019-10-04

**Authors:** Naoki Ozato, Shinichiro Saito, Tohru Yamaguchi, Mitsuhiro Katashima, Itoyo Tokuda, Kaori Sawada, Yoshihisa Katsuragi, Masanori Kakuta, Seiya Imoto, Kazushige Ihara, Shigeyuki Nakaji

**Affiliations:** 1 Department of Active Life Promotion Sciences, Graduate School of Medicine, Hirosaki University, Aomori, Japan; 2 Health Care Food Research Laboratories, Kao Corporation, Tokyo, Japan; 3 Biological Science Research Laboratories, Kao Corporation, Tokyo, Japan; 4 Department of Social Medicine, Graduate School of Medicine, Hirosaki University, Aomori, Japan; 5 Human Genome Center, Institute of Medical Science, University of Tokyo, Tokyo, Japan; 6 Health Intelligence Center, Institute of Medical Science, University of Tokyo, Tokyo, Japan

**Keywords:** Microbiota, Policy and public health in microbiology

## Abstract

The gut microbiota is reported to be related to obesity, and visceral fat is reported to be strongly associated with cardiovascular disease and overall mortality. However, the association between the gut microbiota and obesity has mainly been studied using body mass index (BMI) as a proxy for obesity. We investigated the relationship of both visceral fat and BMI with the gut microbiota stratified by sex in a population-based cross-sectional study of Japanese men and women 20–76 years of age (*n* = 1001). Women with a higher visceral fat area (VFA) harboured a higher relative abundance of the Firmicutes phylum (*P* for trend <0.001) and a lower relative abundance of the Bacteroidetes phylum (*P* for trend 0.030), whereas men with higher VFA harboured a lower relative abundance of the Firmicutes phylum (*P* for trend 0.076) and a higher relative abundance of the Bacteroidetes phylum (*P* for trend 0.013). Similar results were obtained using BMI as an index, but the differences were not significant in men. At the genus level, *Blautia* was the only gut microbe significantly and inversely associated with VFA regardless of sex. In conclusion, at the genus level we found that *Blautia* was the only gut microbe significantly and inversely associated with VFA, regardless of sex.

## Introduction

The gut microbiota is highly involved in host energy regulation and homoeostasis, thereby contributing to diabetes and obesity status.^1^ Some studies report that the gut microbiota, referred to as a “super organism” and a “virtual organ”, may cause obesity.^2–5^ Moreover, the gut microbiota is reported to be highly associated with environmental factors such as dietary habits,^6–8^ age,^9,10^ and sex.^11,12^

Animal studies have revealed that the gut microbiota composition is associated with increased adiposity and insulin resistance.^13,14^ In some human studies, participants classified as obese based on body mass index (BMI) harboured a significantly lower relative abundance of Bacteroidetes compared to non-obese participants, but the studies were performed mostly in women.^15,16^ Other studies, however, revealed conflicting findings. Some studies reported a greater relative abundance of Bacteroidetes in obese participants compared to lean controls,^17,18^ whereas other studies found no correlation between human obesity and the relative abundance of Bacteroidetes or Firmicutes.^19–21^ Studies in which obesity was associated with a lower relative abundance of Bacteroidetes^15,16,22^ were performed mostly in women or female-focused participants, and the effect seemed to disappear when the number of male participants increased.^17–21^ Therefore, we hypothesized that the inconsistent results among studies of the relation between the gut microbiota and excess weight are due to sex differences. In addition to sex differences, the samples in previous studies included fewer than 300 participants,^18^ which could also lead to inconsistent findings. Furthermore, previous studies investigated the association between the gut microbiota and obesity defined on the basis of BMI alone, and not visceral fat area (VFA).

Visceral fat accumulation is a widely recognized risk factor for mortality, likely independent of subcutaneous fat and waist circumference (WC).^23–25^ Clustering of metabolic risk factors, such as hypertension, high blood glucose and triglyceride concentrations, and low serum high-density lipoprotein cholesterol, is more strongly associated with VFA than high subcutaneous fat, WC, or BMI.^26–28^ Reducing VFA could decrease the risk of metabolic syndrome-related disease. Thus, the association between the gut microbiota and VFA might be more important clinically than the association between the gut microbiota and BMI, especially with regard to metabolic syndrome-related disease.

It is well known that the gut flora composition fluctuates with age.^9,10^ In particular, the gut microbiota fluctuates rapidly in older people.^9,10,29^ In the present study, we enrolled Japanese participant up to 76 years of age. We investigated the association between the gut microbiota and both BMI and VFA measured using an impedance method, which provides results that highly correlate with those obtained using computed tomography (*R* > 0.8).^30^

## Results

### Characteristics of participants

**Fig. 1:**
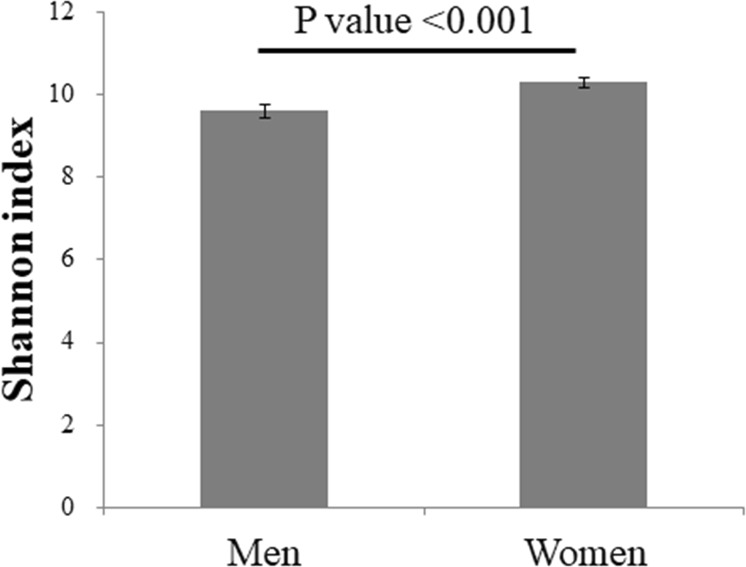
The Shannon index of the gut microbiota stratified by sex. Data are presented as the mean ± standard error. The *P* value was calculated using the Wilcoxon rank-sum test

As a previous study reported a significant difference in the gut microbiota between men and women,^31^ the participants were divided into two groups stratified by sex. The characteristics of the participants stratified by sex are shown in Table 1. Mean age was 51.2 ± 14.1 years for men and 54.2 ± 13.7 years for women. Most of the values were significantly higher in men than in women, including VFA, BMI, WC, and systolic blood pressure. The proportion of overweight (defined as 25 ≤ BMI < 30) was 25.6% for men and 15.8% for women, and the proportion of obesity (defined as BMI ≥ 30) was 4.3% for men and 3.0% for women. These proportions are comparable with those from the 2010 national survey reported by the Japanese government (overweight and obesity rates in those aged 30–69 years: 33.5% for men and 20.5% for women).^32^ Compared to the women, the men drank more alcohol, smoked more, and slept more. Mean VFA values were 68.2 ± 33.3 cm^2^ for women and 106.9 ± 44.9 cm^2^ for men. The Japan Society for the Study of Obesity defines visceral obesity as VFA ≥ 100 cm^2^. The mean frequency of a person taking one or more medications a day (e.g., medicine for hypertension, hyperlipidaemia, diabetes, rheumatism, dementia, or allergy) was 29% in men and 31% in women. The frequency of a person taking diabetes medicine was significantly higher in men than in women. Men consumed significantly more energy than women. Total dietary fibre intake, however, was not significantly different between men and women. In the present study, compared to women, men consumed a significantly higher proportion of carbohydrate and a lower proportion of fat and protein. Gut bacteria were significantly more diverse in women than in men (Fig. 1). The gut microbiota diversity among all participants did not significantly differ by age after adjusting for total dietary fibre intake, smoking habit, alcohol intake, and habitual medicine use.

**Table 1:** Participant characteristics stratified by sex

Characteristics	Sex		*P* value
	Men	Women	
Visceral fat area (cm^2^)^*1^	106.9 ± 44.9	68.2 ± 33.3	<0.001**
Number	391	610	
Age (y)^a^	51.2 ± 14.1	54.2 ± 13.7	0.001**
Height (cm)^a^	169.1 ± 6.6	155.6 ± 6.1	<0.001**
Body weight (kg)^a^	67.9 ± 10.6	54.1 ± 8.7	<0.001**
Body mass index (kg/m^2^)^a^	23.7 ± 3.2	22.4 ± 3.6	<0.001**
Waist circumference (cm)^a^	82.9 ± 8.6	74.2 ± 9.4	<0.001**
Serum glucose (mg/dL)^a^	4.7 ± 1.0	4.5 ± 0.7	0.026*
HbA1c (%)^a^	5.8 ± 0.7	5.8 ± 0.5	0.025*
SBP (mmHg)^a^	125.2 ± 16.3	119.7 ± 17.8	<0.001**
DBP (mmHg)^a^	78.5 ± 11.6	73.0 ± 11.3	<0.001**
Triglyceride (mg/dL)^a^	1.4 ± 1.2	0.9 ± 0.5	<0.001**
Total cholesterol (mg/dL)^a^	5.3 ± 0.8	5.4 ± 0.9	0.152
HDL cholesterol (mg/dL)^a^	1.6 ± 0.4	1.8 ± 0.4	<0.001**
LDL cholesterol (mg/dL)^a^	3.0 ± 0.7	3.1 ± 0.8	0.569
Smoking habit (stick/d)^a^	11.8 ± 11.7	2.3 ± 5.5	<0.001**
Sleep time (h/d)^a^	7.1 ± 1.2	6.8 ± 1.0	<0.001**
Walk speed (s/10 m)^a^	3.6 ± 0.8	4.1 ± 0.8	<0.001**
Habitual medicine use (% of Yes)^b^	28.9%	30.5%	0.641
Hypertension^b^	23.8%	23.1%	0.867
Hyperlipidaemia^b^	9.5%	12.5%	0.144
Diabetes^b^	5.3%	2.0%	0.006**
Rheumatism^b^	0.8%	2.1%	0.347
Total energy intake (kcal/d)^a^	2112.3 ± 579.6	1666.6 ± 454.5	<0.001**
Carbohydrate intake^a^	0.60 ± 0.07	0.57 ± 0.07	<0.001**
Fat intake^a^	0.25 ± 0.06	0.27 ± 0.05	<0.001**
Protein intake^a^	0.15 ± 0.03	0.16 ± 0.03	<0.001**
Alcohol intake (g/d)^a^	21.6 ± 23.4	4.3 ± 10.4	<0.001**
Total dietary fibre intake (g/d)^a^	11.1 ± 4.6	10.8 ± 4.3	0.740

Means ± standard deviations are presented for continuous variables

*P* < 0.05 and < 0.01 are indicated by * and **, respectively

^a^Wilcoxon rank-sum test was used

^b^Test for equality of proportions was used

Furthermore, the characteristics of the participants according to VFA (cut-off point 100 cm^2^) in each sex are shown in Supplementary Table 1 and Supplementary Fig. 1. The gut microbiota only tended to be less diverse in men with higher VFA than in men with lower VFA.

### Sex differences in human gut microbial phyla and metabolic factors

**Fig. 2:**
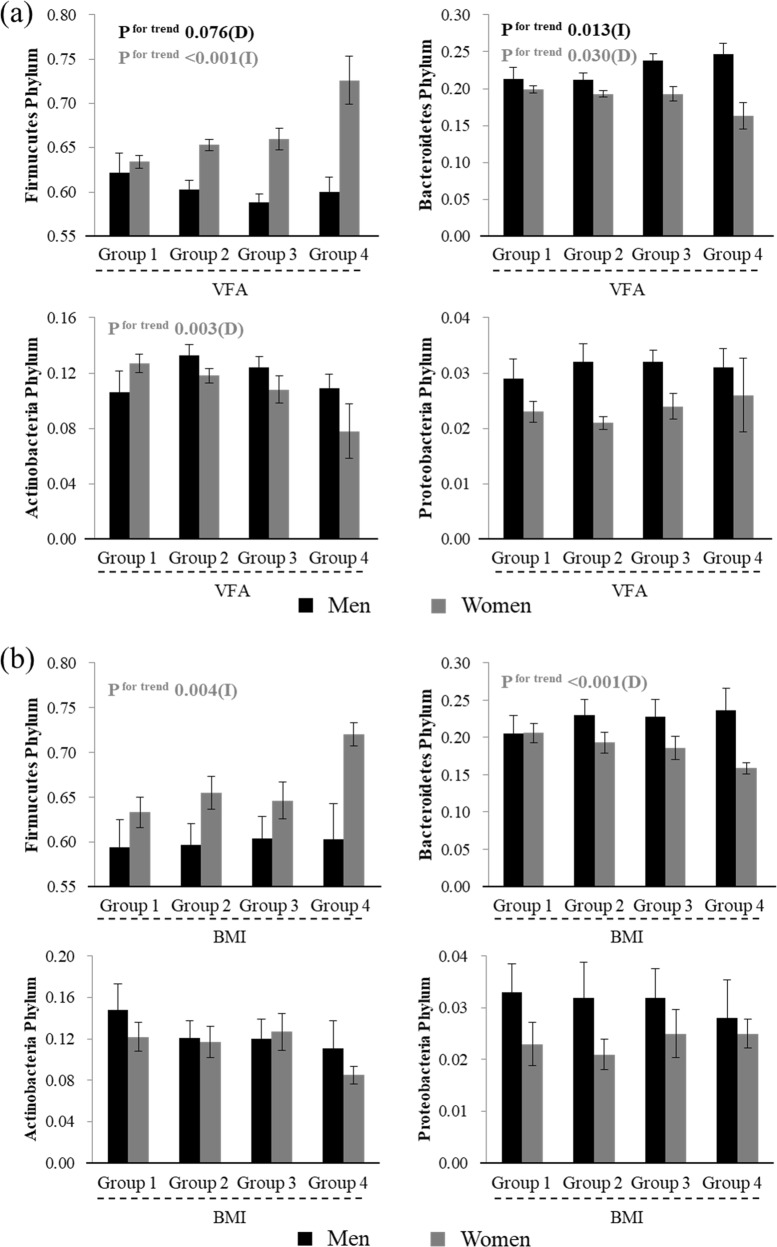
Relationship between VFA or BMI and relative abundance of the four phyla (Firmicutes, Bacteroidetes, Actinobacteria, and Proteobacteria). Black bars represent men and grey bars represent women. Black font indicates significance for men and grey font indicates significance for women. **a** Relationship between VFA and the relative abundance of the four phyla, according to VFA, Group 1: VFA < 50 (*n* = 38 for men and 197 for women), Group 2: 50 ≤ VFA < 100 (*n* = 145 for men and 311 for women), Group 3: 100 ≤ VFA < 150 (*n* = 140 for men and 88 for women), Group 4: 150 ≤ VFA (*n* = 38 for men and 14 for women). **b** Relationship between BMI and the relative abundance of the four phyla, according to BMI, Group 1: BMI < 20 (*n* = 38 for men and 171 for women), Group 2: 20 ≤ BMI < 25 (*n* = 238 for men and 325 for women), Group 3: 25≤ BMI < 30 (*n* = 98 for men and 94 for women), Group 4: 30 ≤ BMI (*n* = 17 for men and 20 for women). Data are presented as the mean ± standard error. *P* values were calculated using the Jonckheere test. (D) indicates tendency to decrease, (I) indicates tendency to increase in relation to VFA

Four phyla, Firmicutes, Bacteroidetes, Actinobacteria, and Proteobacteria, account for the majority (~98%) of the human gut phyla. Figure 2 shows the association between BMI and VFA and the relative abundance of these four dominant phyla. Participants were divided into four groups stratified by their BMI: BMI < 20, 20 ≤ BMI < 25, 25 ≤ BMI < 30, and BMI ≥ 30. Women with higher BMI harboured a significantly higher relative abundance of the Firmicutes phylum (*P* for trend: 0.004), and a significantly lower relative abundance of the Bacteroidetes phylum (*P* for trend: <0.001). No significant associations between these phyla and BMI, however, were observed in men, as reported previously.^19–21^

To statistically evaluate the trend association between VFA and these phyla, we divided the participants into four groups according to their VFA: VFA < 50, 50 ≤ VFA < 100, 100 ≤ VFA < 150, and VFA ≥ 150. Men with a higher VFA tended to harbour a lower relative abundance of the Firmicutes phylum (*P* for trend: 0.076) and a significantly higher relative abundance of the Bacteroidetes phylum (*P* for trend: 0.013). Conversely, women with higher VFA harboured a significantly higher relative abundance of the Firmicutes phylum (*P* for trend: <0.001) and a significantly lower relative abundance of the Bacteroidetes phylum (*P* for trend: 0.030). With regard to the other two phyla, women with higher VFA harboured a lower relative abundance of the Actinobacteria phylum (*P* for trend: 0.003), whereas men with higher VFA did not. Furthermore, VFA was not associated with the relative abundance of the Proteobacteria phylum in either sex.

The effect of sex on the association between VFA and the Firmicutes or Bacteroidetes phylum was further analysed by Spearman’s correlation coefficient stratified by sex. As for Firmicutes, there was a significant difference by sex (*r* = −0.064 for men and *r* = 0.114 for women; test for difference of correlation coefficients, *P* value = 0.006). A similar result was found for Bacteroidetes (*r* = 0.122 for men and *r* = −0.079 for women; test for difference of correlation coefficients, *P* value = 0.002).

Furthermore, there were differences in lifestyle habits, such as smoking habit and alcohol intake, between the sexes; therefore, the *P* values of the associations were adjusted for these confounding factors. In men, the relative abundance of the Firmicutes phylum was not significantly related to VFA, but the relative abundance of the Bacteroidetes phylum was significantly and positively associated with VFA (*P* values = 0.703 and 0.020, respectively). In women, the relative abundance of the Firmicutes phylum was significantly and positively associated with VFA, but the relative abundance of the Bacteroidetes phylum was not significantly associated with VFA (*P* values = 0.013 and 0.178, respectively). These findings suggest that the association between VFA and relative abundance of the Firmicutes or Bacteroidetes phylum tends to differ between sexes.

### Microbial genus and VFA

The relative abundance of two phyla, Firmicutes and Bacteroidetes, was associated with the sex and obesity status on the basis of VFA. Therefore, we investigated whether the microbial genera at the genus level were significantly associated with VFA stratified by sex. Among the 305 genera detected, the changes in the relative abundance of microbial genera comprising <10^−2^% of the abundance ratio were too small to accurately detect and these genera were excluded from the analyses. Thus, 54 genera were examined, and among them 10 genera were significantly associated with VFA in men or women (Table 2, Supplementary Table 2 provides the overall data). Three genera were significantly associated with VFA in men. Men with higher VFA harboured a smaller relative abundance of *Blautia* and *Bifidobacterium* (*P* for trend: 0.003 and 0.021, respectively), and a greater relative abundance of *Prevotella* (*P* for trend: 0.003). In women, nine genera were significantly associated with VFA. Women with a higher VFA harboured a lower relative abundance of *Blautia, Bifidobacterium, Eggerthella, Sutterella*, and *Erysipelotrichaceae incertae sedis* (*P* for trend: 0.020, 0.002, 0.004, 0.008, and 0.003, respectively), and a greater relative abundance of *Clostridium sensu stricto, Roseburia, Ruminococcu*s, and *Megasphaera* (*P* for trend: <0.001, 0.010, 0.006, and 0.041, respectively). Of these, only two genera, *Blautia* and *Bifidobacterium*, showed common trends with VFA within a 20% false discovery rate (FDR) in both sexes. *Blautia* and *Bifidobacterium* comprised 6–8% and 4–8% as the whole of individuals, respectively, and were the relatively dominant microbial genera in the human gut (Table 2). The results of pairwise correlation tests between visceral fat and each microbial genus are shown in Supplementary Table 3. *Blautia* was significantly and inversely correlated with visceral fat in both sexes (*r* = −0.154, *P* value = 0.002 for men and *r* = −0.121, *P* value = 0.003 for women). *Bifidobacterium* was significantly and inversely correlated with visceral fat in both sexes (*r* = −0.135, *P* value = 0.008 for men and *r* = −0.104, *P* value = 0.010 for women).

**Table 2:** Association of the relative abundance of the human gut microbial genera and VFA in men and women

Characteristics	Men					
Group	VFA < 50	50 ≤ VFA < 100	100 ≤ VFA < 150	150 ≤ VFA	*P* for trend^a^	*Q* value^b^
Number	38	145	140	68		
Genus						
Blautia	0.0757 ± 0.0424	0.0761 ± 0.0371	0.0657 ± 0.0344	0.0665 ± 0.0426	0.003(D)**	0.052**
Bifidobacterium	0.0696 ± 0.0903	0.0746 ± 0.0768	0.0655 ± 0.0815	0.0495 ± 0.0584	0.020(D)*	0.186*
Prevotella	0.0534 ± 0.0905	0.0671 ± 0.119	0.0880 ± 0.135	0.113 ± 0.142	0.003(I)**	0.052**
Eggerthella	1.73e−03 ± 3.13e−03	1.48e−03 ± 2.23e−03	1.23e−03 ± 1.77e−03	1.84e−03 ± 4.14e−03	0.196(D)	0.496
Sutterella	0.0148 ± 0.0166	0.0139 ± 0.0161	0.0163 ± 0.0176	0.0133 ± 0.0161	0.395(I)	0.508
Clostridium sensu stricto	2.09e−03 ± 6.03e−03	2.26e−03 ± 4.65e−03	2.79e−03 ± 8.74e–03	2.35e−03 ± 6.54e−03	0.154(I)	0.496
Roseburia	0.0426 ± 0.0448	0.0440 ± 0.0379	0.0357 ± 0.0319	0.0441 ± 0.0442	0.272(D)	0.496
Ruminococcus	0.0267 ± 0.0393	0.0241 ± 0.0356	0.0201 ± 0.0352	0.0280 ± 0.0383	0.338(I)	0.496
Megasphaera	4.45e−03 ± 1.02e−02	4.82e−03 ± 1.16e−02	4.88e−03 ± 1.05e−02	6.52e−03 ± 1.33e−02	0.227(I)	0.496
Erysipelotrichaceae incertae sedis	0.00565 ± 0.0157	0.00286 ± 0.00754	0.00260 ± 0.00621	0.00282 ± 0.00622	0.093(D)	0.387
Characteristics	Women					
Number	197	311	88	14		
Genus						
Blautia	0.0745 ± 0.0368	0.0708 ± 0.0401	0.0661 ± 0.0273	0.0583 ± 0.0239	0.020(D)*	0.104*
Bifidobacterium	0.0806 ± 0.0759	0.0712 ± 0.0752	0.0670 ± 0.0780	0.0355 ± 0.0337	0.002(D)**	0.033**
Prevotella	0.0330 ± 0.0789	0.0365 ± 0.0810	0.0372 ± 0.0799	0.0305 ± 0.0732	0.482(D)	0.503
Eggerthella	2.44e−03 ± 3.34e−03	2.46e−03 ± 4.47e−03	1.31e−03 ± 1.77e−03	9.09e−04 ± 1.51e−03	0.004(D)**	0.038**
Sutterella	9.51e−03 ± 1.52e−02	5.80e−03 ± 9.37e−03	6.79e−03 ± 1.13e−02	3.21e−03 ± 4.79e−03	0.008(D)**	0.062**
Clostridium sensu stricto	1.41e−03 ± 5.32e−03	2.41e−03 ± 5.50e−03	3.16e−03 ± 7.61e−03	5.35e−03 ± 7.87e−03	0.001(I)**	0.019**
Roseburia	0.0372 ± 0.0342	0.0440 ± 0.0416	0.0443 ± 0.0356	0.0646 ± 0.0496	0.010(I)**	0.067**
Ruminococcus	0.0332 ± 0.0449	0.0399 ± 0.0460	0.0410 ± 0.0466	0.0623 ± 0.0560	0.006(I)**	0.047**
Megasphaera	0.00205 ± 0.00856	0.00301 ± 0.00912	0.00250 ± 0.00626	0.000122 ± 0.000297	0.041(I)*	0.157*
Erysipelotrichaceae incertae sedis	0.00485 ± 0.0118	0.00327 ± 0.00720	0.00196 ± 0.00455	0.00112 ± 0.00275	0.003(D)**	0.038**

Only human gut microbial genera significantly associated with VFA are shown

Means ± standard deviations are presented for continuous variables

(D) indicates tendency to decrease, (I) indicates tendency to increase in relation to VFA

*P* < 0.05 and < 0.01 are indicated by * and **, respectively

*Q* < 0.20 and < 0.10 are indicated by * and **, respectively

^a^Jonckheere test was used

^b^*Q* values were derived on the basis of the false discovery rate (FDR)

### Adjusted association between VFA and microbial genus

The human gut microbial composition is affected by a variety of environmental factors, including age^9,10^ and dietary habits.^6–8^ These confounding variables might affect the conclusion. Therefore, the *P* values of the associations were adjusted for the confounding factors. Model 1 includes an adjustment for age (Table 3). Model 2 includes adjustments for smoking habit, alcohol intake, dietary fibre intake, and habitual medicine use. Model 3 includes adjustments for all of the factors in Models 1 and 2, as well as WC and BMI. The *P* values for the trend in each model for *Blautia* were 0.020, 0.038, and 0.038, respectively, with a decrease in men, and all *P* values were 0.004 with a decrease in women. These findings suggested that *Blautia* was significantly and inversely associated with VFA in both sexes, even after adjusting for potential related factors. The *P* values for trend in each model for *Bifidobacterium* were 0.031, 0.012, and 0.012, respectively, with a decrease in men, and 0.069, 0.131, and 0.127, respectively, with a decrease in women. *Bifidobacterium* was significantly and inversely associated with VFA only in men after adjusting for potential related factors. Taken together, these results suggested that only *Blautia* was associated with VFA, independent of the related factors.

**Table 3:** Association between VFA and relative abundance of a microbial genus (Blautia and Bifidobacterium) adjusted by Models 1–3

	Blautia and VFA		Bifidobacterium and VFA	
	Men	Women	Men	Women
	*P* value^a^	*P* value^a^	*P* value^a^	*P* value^a^
Model 1				
Age	0.020(D)*	0.004(D)**	0.031(D)*	0.069(D)
Model 2				
Smoking habit				
Total dietary fibre intake	0.038(D)*	0.004(D)**	0.012(D)*	0.131(D)
Alcohol intake				
Habitual medicine use				
Model 3(1 × 2 + BMI + WC)				
Age				
BMI				
Waist circumference				
Smoking habit	0.038(D)*	0.004(D)**	0.012(D)*	0.127(D)
Total dietary fibre intake				
Alcohol intake				
Habitual medicine use				

(D) indicates tendency to decrease, (I) indicates tendency to increase relative to VFA

*P* < 0.05 and <0.01 are indicated by * and **, respectively

^a^Analysis of variance for linear regression model was used

Table 4 shows the adjusted associations between BMI and *Blautia* or *Bifidobacterium* by age, WC, VFA, smoking habit, alcohol intake, dietary fibre intake, and habitual medicine use. *Blautia* was significantly and inversely associated with BMI in women (*P* for trend: 0.004, 0.002, and 0.002, respectively, for all models), but not in men (*P* for trend: 0.141 and 0.140 for Models 2 and 3, respectively) after adjusting for related factors. *Bifidobacterium* was also significantly and inversely associated with BMI in men (*P* for trend: 0.031, 0.003, and 0.003, respectively, for each model, respectively), but not in women (*P* for trend: 0.486, 0.689, and 0.685 for each model, respectively) after adjusting for related factors.

**Table 4:** Association between BMI and relative abundance of a microbial genus (Blautia and Bifidobacterium) adjusted by Models 1–3

	Blautia and BMI		Bifidobacterium and BMI	
	Men	Women	Men	Women
	*P* value^a^	*P* value^a^	*P* value^a^	*P* value^a^
Model 1				
Age	0.020(D)*	0.004(D)**	0.031(D)*	0.486(D)
Model 2				
Smoking habit				
Total dietary fibre intake	0.141(D)	0.002(D)**	0.003(D)**	0.689(D)
Alcohol intake				
Habitual medicine use				
Model 3(1 × 2 + VFA + WC)				
Age				
VFA				
Waist circumference				
Smoking habit	0.140(D)	0.002(D)**	0.003(D)**	0.685(D)
Total dietary fibre intake				
Alcohol intake				
Habitual medicine use				

(D) indicates tendency to decrease, (I) indicates tendency to increase relative to VFA

*P* < 0.05 and < 0.01 are indicated by * and **, respectively

^a^Analysis of variance for linear regression model was used

In addition, multiple linear regression analysis was performed with *Blautia* as the objective variable. The explanatory variables were age and body composition (BMI, WC, body fat percentage, and VFA), lifestyle habits (sleep time, physical activity, smoking habit, and habitual medicine use), and dietary habits (daily intake of carbohydrate, protein, fat, alcohol, and dietary fibre). As shown in Supplementary Table 4, a stepwise analysis was performed by multiple regression analysis. Two variables (VFA and age) for men and five variables (VFA, age, sleep time, walking speed, and smoking habit) for women remained related to *Blautia*. Among them, only VFA was significantly and inversely associated with *Blautia* (*P* value 0.032 for men and 0.015 for women). These results suggest that VFA is the only explanatory variable among the related variables associated with *Blautia* in both sexes, supporting the results shown in Table 3.

### Confirmation group analysis

To confirm the reproducibility of the association between *Blautia* and VFA, a total of 326 individuals who participated in the 2016 health check, but not in the 2015 health check, were enrolled as a confirmation group. The characteristics of the confirmation group were similar to those of the main study group (Supplementary Table 5). The results of the analyses on *Blautia* and VFA of the confirmation group were similar to those of the main study sample; *Blautia* was significantly and inversely associated with VFA (*P* for trend 0.049), and the *P* value after adjusting for the related factors in Model 3 and sex was 0.038.

## Discussion

This study reports a sex-dependent association between VFA and the gut microbiota in a relatively large number of participants (1001 Japanese individuals). Ethnicity is a major factor shaping the composition of the gut microbiota.^33^ The relative abundance of the four major phyla in the gut, Firmicutes, Bacteroidetes, Actinobacteria, and Proteobacteria, was similar to that reported in a previous study of a Japanese population.^34^ In other populations, such as in the USA, China, and Russia, the relative abundance of the Firmicutes and Bacteroidetes phyla was similar, but the relative abundance of Actinobacteria was lower and that of Proteobacteria was higher compared to a Japanese population.^34^ The gut microbiota is reported to be less diverse in obese individuals than in non-obese individuals.^18^ In the present study, we found that the gut microbiota had significantly higher diversity in women than in men (Fig. 1). This may explain, in part, the higher obesity rate in men than in women in the present study.

We found that women with higher VFA harboured a higher relative abundance of the Firmicutes phylum and a lower relative abundance of the Bacteroidetes phylum, whereas the opposite was found in men with higher VFA: a lower relative abundance of the Firmicutes phylum and higher relative abundance of the Bacteroidetes phylum. Similar results were obtained when using BMI as an index of obesity, but the differences in men were not significant. These findings suggest that the relative abundance of the two phyla, Bacteroidetes and Firmicutes, tended to differ depending on BMI and sex. In previous studies, the association between these two phyla and obesity was inconsistent.^3,15,17–22^ Our hypothesis that the inconsistency of the findings of the previous studies is due to sex differences is supported by the findings of the present study, although intervention trials are needed to further test the hypothesis. Furthermore, the relative abundance of the two phyla differed in association with sex and obesity on the basis of VFA, which, compared to BMI, more strongly associates with cardiovascular disease and overall mortality.^26,27^ These associations between the two phyla and VFA have not been reported previously and suggest the importance of distinguishing between men and women, and measuring VFA instead of BMI when evaluating the association between obesity and gut microbiota.

At the genus level, we observed that only one microbial genus, *Blautia*, was significantly associated with VFA, regardless of sex. After adjusting for related factors, such as age, BMI, WC, dietary fibre intake, smoking habit, alcohol intake, and habitual medicine use, the relative abundance of *Blautia* was significantly associated with VFA independent of sex. The relative abundance of *Bifidobacterium* was significantly associated with VFA, but only in men. Even after yoghurt intake was added as one of the confounding factors used in Model 3, the relative abundance of *Bifidobacterium* and visceral fat remained significantly and inversely associated. Taken together, only the relative abundance of *Blautia* was significantly and inversely associated with VFA independent of BMI or WC, regardless of sex. Furthermore, although the relative abundance of *Erysipelotrichaceae incertae sedis* was significantly associated with BMI but not with VFA regardless of sex, only that of *Bifidobacterium* and *Blautia* was significantly associated with VFA but not with BMI, suggesting that different gut microbes are associated with BMI and VFA.

Diet^6–8^ and age^9,10^ have a great effect on microbial communities, therefore we included these two factors in the analysis to evaluate the associations between age and dietary habits, and the relative abundance of *Blautia* and/or *Bifidobacterium*. The relative abundance of *Blautia* was not significantly correlated with age, whereas the relative abundance of *Bifidobacterium* was significantly and inversely correlated with age. Thus, age might be one reason that the relative abundance of *Bifidobacterium* was not significantly associated with VFA in women after adjusting for age. Dietary factors (protein, fat, carbohydrate, and dietary fibre) were adopted as explanatory factors in the multiple regression model), but none of these factors was associated with the relative abundance of *Blautia*.

*Blautia* is significantly and positively associated with visceral fat mass estimated by dual-x-ray absorptiometry in older people (mean age, 63 years).^35^ Furthermore, because the method for estimating visceral fat mass was not described in the previous report,^35^ the specific method for distinguishing visceral fat from subcutaneous fat mass using dual-x-ray absorptiometry was unclear.

Obesity (assessed using BMI as an index) improves following the ingestion of *Bifidobacterium*.^36^ In the present study, however, the relative abundance of *Bifidobacterium* was significantly and inversely associated with BMI and VFA in men, but not in women after adjusting for related factors, such as age, BMI, WC, dietary fibre intake, smoking habit, alcohol intake, and habitual medicine use. These findings suggest that the association between the relative abundance of *Bifidobacterium* and BMI or VFA is sex dependent. Furthermore, although a previous study reported that the relative abundance of *Ruminococcus* is significantly higher in obese people on the basis of BMI as an index of obesity,^17^ in the present study it was significantly and positively associated with VFA in women but not in men, suggesting that the association between *Ruminococcus* and VFA is also sex dependent. A previous study reported that a higher *Prevotella*/*Bacteroides* ratio improves glucose metabolism in subjects with a high dietary fibre intake,^37^ but in our study, the relative abundance of *Prevotella* was significantly and positively associated with VFA in men even after adjusting for the factors in Model 3, including intake of total dietary fibre; thus, further studies are needed.

*Blautia* is a gut microbial genus that produces butyric acid and acetic acid,^38^ which decrease obesity by regulating G-protein coupled receptors (GPR) 41 and 43.^39,40^ Additionally, *Blautia* is one of the most abundant genera in the gut, regardless of race,^34,41,42^ but its relative abundance was inversely associated with VFA in the present study. Furthermore, *Blautia* is less prominent in diabetic adults and paediatric patients^43,44^ and those with other diseases, such as liver cirrhosis, rectal cancer, and rheumatoid arthritis.^45,46^ Thus, although further investigation is required to determine whether a change in the relative abundance of *Blautia* in the gut microbiota affects metabolic syndrome risk factors, *Blautia* may have the potential to maintain or improve the status of those diseases related to metabolic syndrome, and could potentially be a new target/index against obesity and diabetes. *Bifidobacterium* is a genus that produces lactic acid and acetic acid, which regulates GPR41 and GPR43.^39,40^ Furthermore, an intervention study demonstrated that intake of *Bifidobacterium* reduces visceral fat.^47,48^

In the present study, we evaluated sex differences in the relationship between visceral fat and the gut microbiota in a relatively large Japanese population. We focused on sex differences, whereas previous reports did not. Statistical adjustments were made for related factors, such as age, BMI, WC, dietary fibre intake, smoking habit, alcohol intake, and habitual medicine use. We found that *Blautia* was an independent microbial genus associated with VFA. We confirmed the reproducibility of our findings in an independent Japanese study population that participated in a health check conducted in a different year.

A limitation of the present study is that it was a cross-sectional study and not a longitudinal cohort study, and we could not assess whether a lower relative abundance of *Blautia* is a risk factor for the future incidence of visceral obesity. Because this study was performed in a limited country, region, and race, reproducibility should be confirmed in a different country and/or race. Our results suggest that, compared to BMI, VFA is more closely associated with microbial flora. *Blautia* was the only microbial genus for which the relative abundance was significantly and inversely associated with visceral fat accumulation independent of BMI and WC in both sexes. Although hierarchical clustering was performed on the basis of the gut microbiota, no feature related to *Blautia* and VFA was detected. Although proton pump inhibitors are reported to change the gut microbiota,^49^ no data on the use of proton pump inhibitors were collected from our study populations. Furthermore, although we collected information on participant use of medicines, we did not obtain information on the antimicrobial effects of the medicines. Lastly, the differences in the results by sex could feasibly be due entirely to sex differences in VFA or in environmental factors; therefore, intervention trials are needed to further test the differences.

In conclusion, at the genus level we found that *Blautia* was the only gut microbial genus that was significantly and inversely associated with VFA, regardless of sex. Our data suggest that clinical trials to evaluate the effects of both sex and *Blautia* might elucidate potential methods for maintaining or improving the status of VFA.

## Methods

### Design, participants, and ethics

The Iwaki Health Promotion Project was launched in 2005 as an annual health check-up for local residents, aiming to prolong a healthy lifespan. Participants were men and women at least 20 years of age living in the Iwaki region of Hirosaki City, Aomori Prefecture, Japan.^50–53^ VFA was first introduced as a health check-up parameter in 2015, and the present analyses were performed using data obtained from the 2015 health check-up as a population-based cross-sectional study. In 2015, 1082 individuals participated in the health check-up. Of these, 12 participants did not complete the clinical assessment and were excluded from the analyses. It is well known that the gut flora fluctuate with age. In particular, the gut microbiota fluctuates rapidly in older people in their 80s.^9,10,29^ In the present study, to minimize age-related diversity in gut microbiota, we enrolled participants <77 years of age. Thus, 1001 individuals (391 men, 610 women; mean age ± standard deviation, 51.2 ± 14.1 years for men, 54.2 ± 13.7 years for women) were enrolled into the study. The only exclusion criteria was age 77 years or older. In addition, a confirmation group of 326 individuals who participated in the 2016 health check, but not in the 2015 health check (62% female, mean age 50.7 ± 17.5 years), was enrolled to confirm the reproducibility of the findings obtained with the 2015 cohort. All of the 2016 participants were under 77 years of age. The study was approved by the Ethics Committee of Hirosaki University School of Medicine and conducted in accordance with the principles of the Declaration of Helsinki (2014-377 and 2016-028). Written informed consent was obtained from all participants prior to the study. This study was registered at the University Hospital Medical Information Network (UMIN-CTR, https://www.umin.ac.jp) prior to the analyses (UMIN ID: UMIN000030351).

### Measurements of the gut microbiota

Faecal samples from each participant were collected using a commercial tube kit (TechnoSuruga Laboratory Co., Ltd., Shizuoka, Japan) and cotton swabs within 3 days prior to the study. Sampling kits were filled with 3 mL of GTC solution (100 mM Tris-HCl (pH 8.0), 40 mM Tris-EDTA (pH 8.0), 4 M guanidine thiocyanate, and 0.001% bromothymol blue), and the cotton swabs were filled with 1 mL of GTC solution. Faecal samples were stored at 4 °C until the DNA was extracted as reported previously.^54–56^

GTC buffer solutions containing faecal samples (800 μL of faeces) were added to tubes filled with zirconium beads. The tubes were then mixed at room temperature for 2 min at a speed of 5 m/s, using a FastPrep 24 Instrument (MP Biomedicals, Santa Ana, CA, USA). After cooling, the samples were centrifuged at 2350  ×  *g* for 1 min. The DNA was then extracted from the bead-treated suspension using an automatic nucleic acid extractor (Precision System Science, Chiba, Japan). A MagDEA DNA 200 (GC) reagent kit (Precision System Science) was used for automatic nucleic acid extraction. The final concentration of each DNA sample was adjusted to 10 ng/μL. We completed extraction of all sample DNA within 4 months. Previous studies reported the stability of samples at 4 °C for 3 weeks.^54^ We confirmed the stability of samples stored for 1 year at 4 °C (Supplementary Fig. 2).

Universal primer sets were used to amplify the V3–V4 region of the prokaryotic 16S rRNA gene, as described previously.^57^ The polymerase chain reaction (PCR) mixture and conditions were performed as described previously.^57^ To check the size of the amplified fragments, 2.0-μL aliquots of the PCR reaction mixtures were electrophoresed on 1.0% agarose gels. The amplified fragments were purified using PCR Cleanup Filter Plates (Merck Millipore, Burlington, MA, USA). The purified PCR fragments were quantified by real-time quantitative PCR (q-PCR) using the methods described by Takahashi et al.^57^ Illumina paired-end sequencing was performed using the 2 × 300 cycle paired-end method on the MiSeqTM system (Illumina, San Diego, CA, USA).

The multiplexed paired-end reads from the Illumina MiSeq system were processed as follows. The adaptor sequences and low-quality bases (threshold = 20) were trimmed at the 3’-end of the reads by Cutadapt (version: 1.13). Reads containing N bases and shorter than 150 bases were discarded. The paired-end reads above the filter threshold were merged to form a single read, called a “merged read”. Merged reads shorter than 370 or longer than 470 were excluded by the fastq_mergepairs subcommand of VSEARCH (version: 2.4.3). Merged reads with more than one expected sequencing error were also excluded. After removing chimera reads detected by the uchime_denovo subcommand of VSEARCH, the remaining merged reads were clustered at a sequence identity ≥97%. The taxa of the identified clusters were predicted by applying RDP Classifier (commit hash: 701e229dde7cbe53d4261301e23459d91615999d) based on their representative reads. Results with a confidence value below 0.8 were treated as unclassified. The proportion of each genus of the gut microbiota is a composition ratio obtained by dividing the number of read counts of each genus by the total number of read counts.

### Measurements of other items

All participants attended a health check-up early in the morning after fasting at least 9 h. The VFA was measured using a visceral fat metre: the EW-FA90 (Panasonic Corporation, Osaka, Japan), which is an authorized medical device in Japan (No. 22500BZX00522000). The results using this device highly correlate with those obtained from computed tomography,^30^ the gold standard for VFA measurement. The following clinical characteristics were also measured: height, body weight, BMI, WC, fasting serum glucose, glycated haemoglobin, systolic blood pressure, diastolic blood pressure, total serum cholesterol concentration, triglycerides, and high-density lipoprotein cholesterol. All laboratory tests were outsourced to LSI Medience Co. (Tokyo, Japan) according to the instructions of the vendors. Blood samples were collected from the peripheral veins of the participants in the morning. Smoking habit (cigarettes/d) and sleep time (h/d) were determined from questionnaires, and walking speed (s/10 m) was investigated using a toe-tip-mounted inertial sensor. Daily intake of protein, fat, carbohydrate, alcohol, and total dietary fibre was calculated from the Brief Diet History Questionnaire.^58,59^

### Statistical analysis

Characteristics of the study participants are reported as means ± standard deviation (SD). Participant groups were compared using the Wilcoxon rank-sum test for two groups and the exact Jonckheere test for trend of more than two groups. Furthermore, when testing the ratio, test for equality of proportions was used. To examine the effect of sex on the association between VFA and the relative abundance of the Firmicutes or Bacteroidetes phylum, we performed a test for differences between correlation coefficients using Spearman’s correlation coefficient. The association was further assessed by analysis of variance for a linear regression model with relative abundance of the individual Firmicutes or Bacteroidetes phylum as an objective variable, and VFA and covariates as explanatory variables. To determine the genera associated with VFA, an FDR correction was used. A 20% FDR (*Q* value < 0.20) was set as the screening cut-off for the following analyses. The relationship between VFA or BMI and the relative abundance of the human gut microbes evaluated was assessed by Spearman’s correlation coefficient or by analysis of variance for a linear regression model with relative abundance of individual genus as an objective variable, and VFA and covariates, such as age and lifestyle habits, as explanatory variables. Multiple regression analysis with stepwise variable selection method was performed to investigate independent explanatory variables for the selected human gut microbes. To validate the linearity of the data, we analysed partial residuals of the fitted multiple linear regression model. We observed no clear bias in the residuals and concluded that the use of linear models was a reasonable choice. Diversity of the gut microbiota (alpha-diversity) was evaluated using the Shannon index. To perform a data reduction analysis on the microbiome composition, hierarchical clustering was performed using Ward’s method.

Statistical tests were two-tailed except for the Jonckheere test for trend. Values of *P* < 0.05 were considered statistically significant. All analyses were performed using the R software version 3.3.4.

### Reporting Summary

Further information on research design is available in the Nature Research Reporting Summary linked to this article.

## Supplementary information

Supplemental materials

Reporting Summary

## Data Availability

All data generated during and analysed during the current study are included in this article and its Supplementary information files, or are available from the corresponding author upon reasonable request.
