# Supplementary material for: Blautia genus associated with visceral fat accumulation in adults 20–76 years of age
Source: NPJ Biofilms Microbiomes. 2019 Oct 4;5:28. doi: 10.1038/s41522-019-0101-x (PMC6778088; doi:10.1038/s41522-019-0101-x)
Supplement: Supplementary file 1 — Supplemental materials [file 41522_2019_101_MOESM1_ESM.pdf]

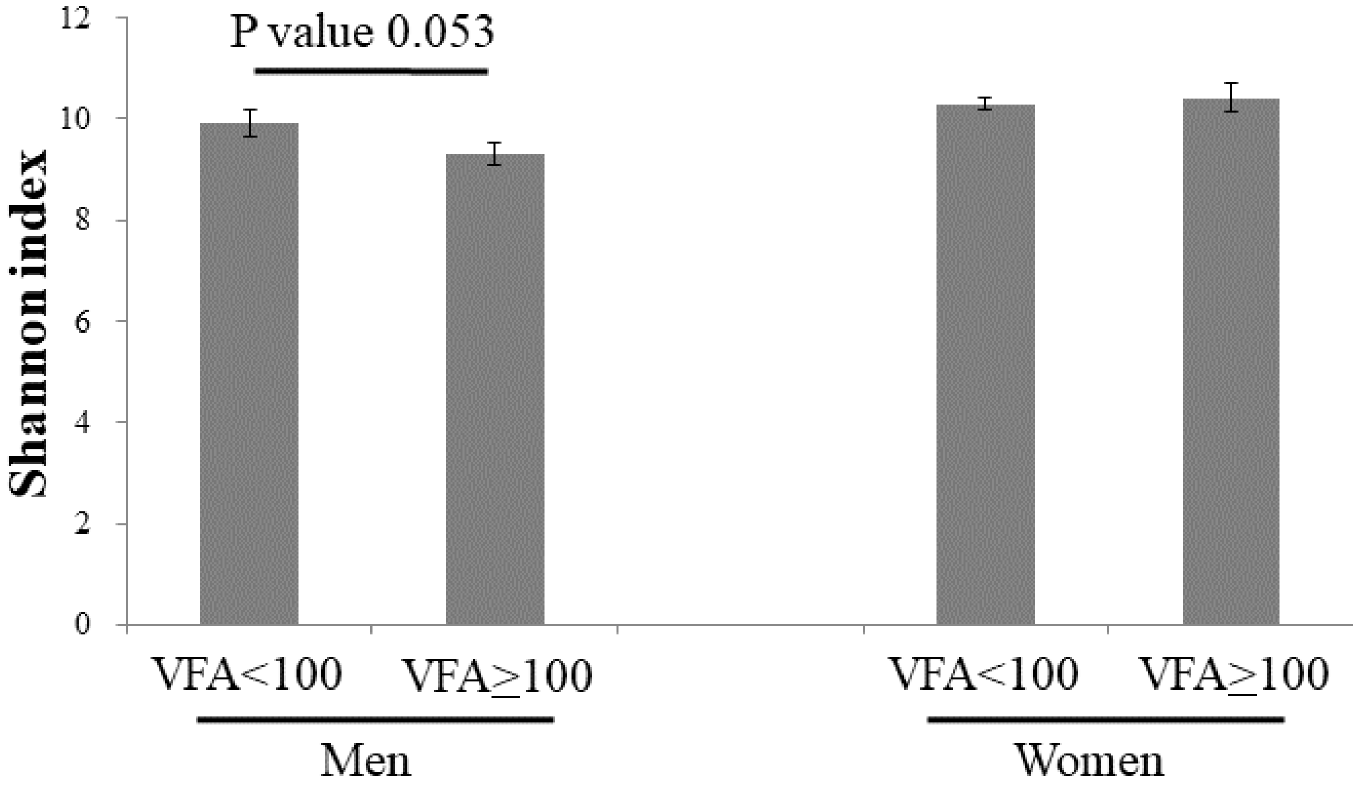

Supplementary Figure 1 The Shannon index of the gut microbiota stratified by VFA in each sex

The data are presented as mean ± standard error. The P-value was calculated using the Wilcoxon rank-sum test.

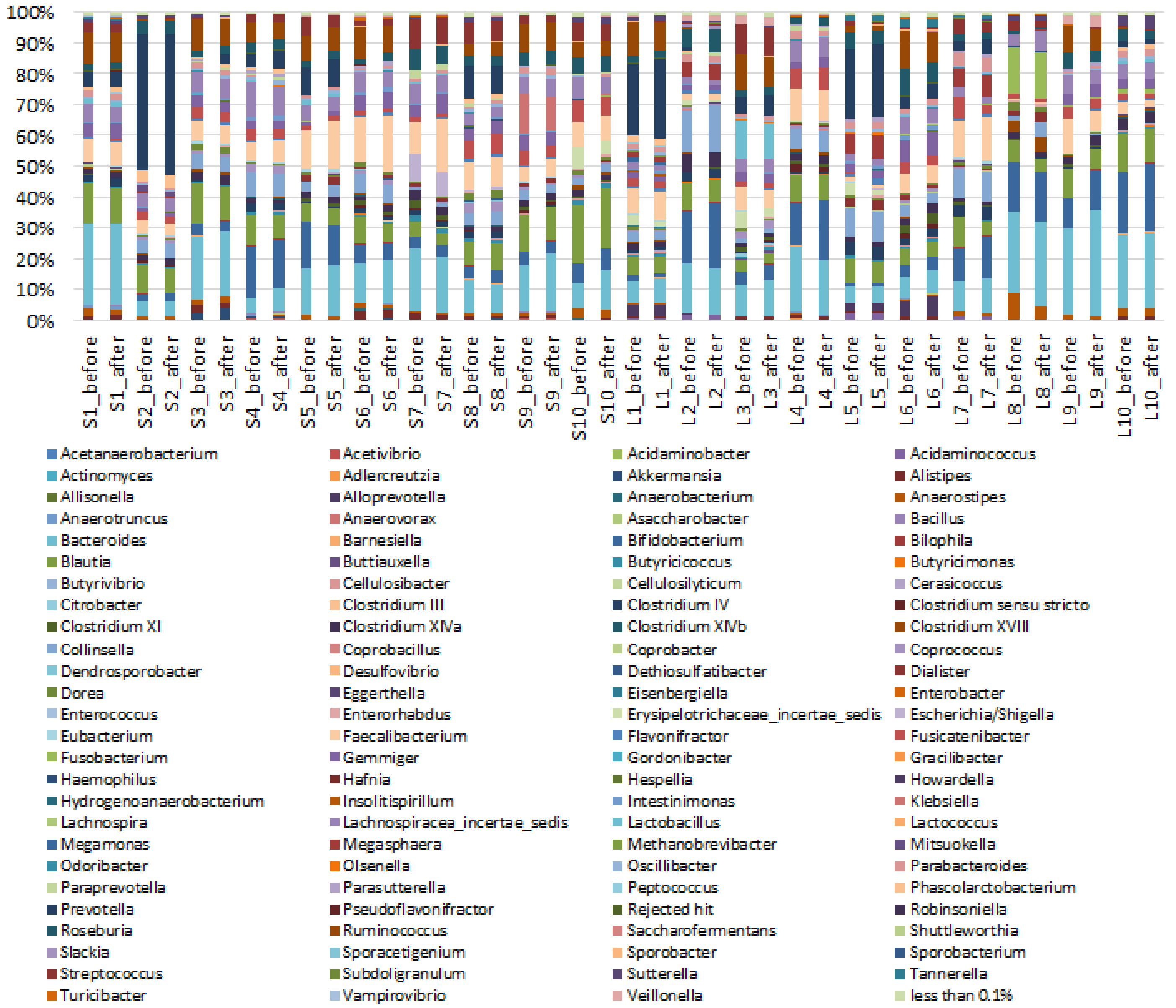

Group 1	Correlation coefficient
S1	0.997
S2	0.999
S3	0.986
S4	0.984
S5	0.983
S6	0.979
S7	0.958
S8	0.989
S9	0.986
S10	0.972

Group 2	Correlation coefficient
L1	0.990
L2	0.984
L3	0.980
L4	0.972
L5	0.992
L6	0.978
L7	0.953
L8	0.979
L9	0.975
L10	0.994

Supplementary Figure 2 Preservation stability of gut microbiota after storing for 1 year at 4°C

Pearson's correlation coefficient was used to evaluate the stability of the samples before and after preservation for 1 year. Group 1 and Group 2 were evaluated at different times. Gut microbiota with a relative abundance of less than 0.1% is described as < 0.1%.

Supplementary Table 1. Characteristics of the subjects in the main study group stratified by sex and VFA

Characteristics	Men		P value	Women		P value
	VFA<100	100≤VFA		VFA<100	100≤VFA	
**Visceral fat area (cm^2)**	69.5 ± 22.8	140.2 ± 31.4		57.5 ± 22.2	124.9 ± 23.4	
**Number**	183	208		513	97	
**Age (y)**[*1]	49.1 ± 14.8	53.1 ± 13.3	0.009**	53.2 ± 13.8	59.4 ± 12.0	<0.001**
**Height (cm)**[*1]	169.2 ± 6.8	169.0 ± 6.5	0.966	155.9 ± 6.0	154.2 ± 6.6	0.015*
**Body weight (kg)**[*1]	62.2 ± 7.0	72.9 ± 10.8	<0.001**	52.0 ± 6.8	65.1 ± 9.1	<0.001**
**Body mass index (kg/m^2)**[*1]	21.7 ± 2.0	25.5 ± 3.0	<0.001**	21.4 ± 2.7	27.4 ± 3.5	<0.001**
**Waist circumference (cm)**[*1]	76.8 ± 5.3	88.3 ± 7.3	<0.001**	71.6 ± 7.2	87.8 ± 8.0	<0.001**
**Serum Glucose (mg/dL)**[*1]	4.4 ± 0.6	4.9 ± 1.2	<0.001**	4.4 ± 0.7	4.8 ± 0.7	<0.001**
**HbA1c (%)**[*1]	5.6 ± 0.4	5.9 ± 0.8	<0.001**	5.7 ± 0.4	6.0 ± 0.6	<0.001**
**SBP (mmHg)**[*1]	122.5 ± 17.0	127.7 ± 15.3	0.001**	117.8 ± 17.2	129.7 ± 17.5	<0.001**
**DBP (mmHg)**[*1]	76.3 ± 12.2	80.3 ± 10.7	<0.001**	72.1 ± 11.1	78.2 ± 11.0	<0.001**
**Triglyceride (mg/dL)**[*1]	1.1 ± 0.7	1.7 ± 1.5	<0.001**	0.9 ± 0.4	1.2 ± 0.6	<0.001**
**Total cholesterol (mg/dL)**[*1]	5.1 ± 0.8	5.5 ± 0.8	<0.001**	5.4 ± 0.9	5.6 ± 0.8	0.005**
**HDL cholesterol (mg/dL)**[*1]	1.7 ± 0.4	1.5 ± 0.4	<0.001**	1.9 ± 0.4	1.6 ± 0.4	<0.001**
**LDL cholesterol (mg/dL)**[*1]	109.6 ± 27.2	123.1 ± 26.8	<0.001**	116.4 ± 29.4	129.0 ± 26.9	<0.001**
**Smoking habit (stick/d)**[*1]	11.0 ± 11.4	12.5 ± 12.0	0.240	2.3 ± 5.4	2.4 ± 5.6	0.741
**Sleep time (h/d)**[*1]	7.1 ± 1.3	7.2 ± 1.2	0.328	6.8 ± 1.0	7.0 ± 1.0	0.039*
**Walk speed (sec/10m)**[*1]	3.6 ± 0.8	3.6 ± 0.7	0.866	4.0 ± 0.7	4.5 ± 1.0	0.001**
**Habitual medicine use (% of Yes)**[*2]	20.1%	36.7%	<0.001**	27.3%	47.4%	<0.001**
**Hypertension**[*2]	14.1%	32.4%	<0.001**	20.3%	38.1%	<0.001**
**Hyperlipidaemia**[*2]	4.9%	13.5%	0.006**	11.3%	18.6%	0.069
**Diabetes**[*2]	2.7%	7.7%	0.049*	1.8%	3.1%	0.637
**Rheumatism**[*2]	1.1%	0.5%	0.918	1.8%	4.1%	0.272
**Total energy intake (kcal/d)**[*1]	8879.8 ± 2597.8	8810.7 ± 2266.7	0.782	6946.2 ± 1920.5	7140.5 ± 1802.1	0.402
**Carbohydrate intake**[*1]	0.60 ± 0.08	0.59 ± 0.07	0.119	0.57 ± 0.07	0.58 ± 0.07	0.042*
**Fat intake**[*1]	0.25 ± 0.06	0.26 ± 0.05	0.153	0.27 ± 0.05	0.26 ± 0.05	0.013*
**Protein intake**[*1]	0.15 ± 0.03	0.15 ± 0.03	0.100	0.16 ± 0.03	0.16 ± 0.03	0.426
**Alcohol consumption (g/d)**[*1]	19.0 ± 21.9	23.9 ± 24.6	0.041*	4.5 ± 10.7	3.3 ± 8.9	0.056
**Total dietary fibre intake (g/d)**[*1]	11.1 ± 4.8	11.0 ± 4.5	0.975	10.8 ± 4.3	10.7 ± 4.1	0.864

P<0.05 and <0.01 are indicated by * and **, respectively.

***1 Wilcoxon rank-sum test was used**

***2 test for equality of proportions was used**

Supplementary Table 2. Relation of the relative abundance of human gut microbial genera and VFA

	MEN							WOMEN						
	VFA <50	50≤ VFA <100	100≤ VFA ≤150	150≤ VFA	P for trend[*1]		Q value[*2]	VFA <50	50≤ VFA <100	100≤ VFA ≤150	150≤ VFA	P for trend[*1]		Q value[*2]
Number	38	145	140	68				197	311	88	14			
Genus														
Acidaminococcus	0.0009 ± 0.0022	0.0013 ± 0.0041	0.0018 ± 0.0043	0.0021 ± 0.0052	0.067	(I)	0.329	0.0012 ± 0.0037	0.0011 ± 0.0033	0.0012 ± 0.0037	0.0015 ± 0.0055	0.454	(D)	0.501
Akkermansia	0.0077 ± 0.0319	0.0088 ± 0.0357	0.0023 ± 0.0127	0.0020 ± 0.0065	0.226	(D)	0.496	0.0063 ± 0.0172	0.0087 ± 0.0287	0.0039 ± 0.0105	0.0016 ± 0.0044	0.098	(D)	0.258
Alistipes	0.0147 ± 0.0221	0.0143 ± 0.0209	0.0124 ± 0.0193	0.0108 ± 0.0122	0.338	(I)	0.496	0.0282 ± 0.0322	0.0263 ± 0.0318	0.0236 ± 0.0289	0.0264 ± 0.0238	0.221	(D)	0.366
Alloprevotella	0.0032 ± 0.0104	0.0042 ± 0.0155	0.0065 ± 0.0208	0.0081 ± 0.0218	0.380	(I)	0.508	0.0022 ± 0.0113	0.0004 ± 0.0032	0.0023 ± 0.0121	0.0001 ± 0.0004	0.493	(D)	0.503
Anaerostipes	0.0470 ± 0.0574	0.0599 ± 0.0612	0.0588 ± 0.0635	0.0457 ± 0.0526	0.423	(D)	0.508	0.0594 ± 0.0561	0.0499 ± 0.0497	0.0555 ± 0.0597	0.0242 ± 0.0188	0.067	(I)	0.238
Bacteroides	0.1171 ± 0.0913	0.1098 ± 0.0708	0.1166 ± 0.0771	0.0998 ± 0.0757	0.250	(D)	0.496	0.1185 ± 0.0692	0.1120 ± 0.0686	0.1147 ± 0.0707	0.0915 ± 0.0628	0.088	(D)	0.257
Barnesiella	0.0011 ± 0.0022	0.0007 ± 0.0015	0.0009 ± 0.0022	0.0008 ± 0.0016	0.442	(D)	0.508	0.0017 ± 0.0029	0.0015 ± 0.0033	0.0010 ± 0.0022	0.0013 ± 0.0015	0.091	(D)	0.257
Bifidobacterium	0.0696 ± 0.0903	0.0746 ± 0.0768	0.0655 ± 0.0815	0.0495 ± 0.0584	0.021*	(D)	0.186*	0.0806 ± 0.0759	0.0712 ± 0.0752	0.0670 ± 0.0780	0.0355 ± 0.0337	0.002**	(D)	0.033**
Bilophila	0.0009 ± 0.0013	0.0013 ± 0.0018	0.0020 ± 0.0030	0.0014 ± 0.0026	0.245	(I)	0.496	0.0018 ± 0.0026	0.0017 ± 0.0025	0.0015 ± 0.0020	0.0019 ± 0.0030	0.490	(D)	0.503
Blautia	0.0757 ± 0.0424	0.0761 ± 0.0371	0.0657 ± 0.0344	0.0665 ± 0.0426	0.003**	(D)	0.052**	0.0745 ± 0.0368	0.0708 ± 0.0401	0.0661 ± 0.0273	0.0583 ± 0.0239	0.020*	(D)	0.104*
Butyricicoccus	0.0060 ± 0.0062	0.0070 ± 0.0047	0.0066 ± 0.0055	0.0069 ± 0.0054	0.435	(D)	0.508	0.0063 ± 0.0049	0.0062 ± 0.0049	0.0065 ± 0.0050	0.0072 ± 0.0029	0.384	(I)	0.469
Catenibacterium	0.0017 ± 0.0065	0.0012 ± 0.0057	0.0054 ± 0.0147	0.0041 ± 0.0174	0.161	(I)	0.496	0.0027 ± 0.0117	0.0016 ± 0.0080	0.0027 ± 0.0095	0.0093 ± 0.0231	0.362	(I)	0.465
Clostridium.IV	0.0220 ± 0.0426	0.0138 ± 0.0222	0.0146 ± 0.0245	0.0198 ± 0.0308	0.256	(I)	0.496	0.0212 ± 0.0251	0.0257 ± 0.0367	0.0249 ± 0.0359	0.0305 ± 0.0414	0.270	(I)	0.404
Clostridium.sensu.stricto	0.0021 ± 0.0060	0.0023 ± 0.0047	0.0028 ± 0.0087	0.0024 ± 0.0065	0.154	(I)	0.496	0.0014 ± 0.0053	0.0024 ± 0.0055	0.0032 ± 0.0076	0.0054 ± 0.0079	<0.001**	(I)	0.019**
Clostridium.XlVa	0.0050 ± 0.0048	0.0043 ± 0.0038	0.0040 ± 0.0030	0.0056 ± 0.0067	0.329	(I)	0.496	0.0057 ± 0.0072	0.0050 ± 0.0040	0.0055 ± 0.0039	0.0042 ± 0.0031	0.216	(I)	0.366
Clostridium.XlVb	0.0016 ± 0.0025	0.0021 ± 0.0029	0.0027 ± 0.0036	0.0025 ± 0.0033	0.016*	(I)	0.173*	0.0023 ± 0.0041	0.0021 ± 0.0037	0.0018 ± 0.0030	0.0031 ± 0.0047	0.075	(D)	0.238
Clostridium.XVIII	0.0070 ± 0.0092	0.0050 ± 0.0067	0.0045 ± 0.0064	0.0057 ± 0.0082	0.483	(I)	0.509	0.0054 ± 0.0059	0.0059 ± 0.0072	0.0057 ± 0.0072	0.0023 ± 0.0017	0.112	(D)	0.258
Collinsella	0.0292 ± 0.0329	0.0512 ± 0.0445	0.0524 ± 0.0397	0.0508 ± 0.0543	0.063	(I)	0.329	0.0392 ± 0.0432	0.0384 ± 0.0413	0.0353 ± 0.0340	0.0358 ± 0.0394	0.409	(D)	0.469
Coprococcus	0.0069 ± 0.0109	0.0070 ± 0.0092	0.0056 ± 0.0070	0.0062 ± 0.0075	0.441	(D)	0.508	0.0065 ± 0.0094	0.0075 ± 0.0112	0.0076 ± 0.0092	0.0148 ± 0.0137	0.040*	(I)	0.156
Dialister	0.0016 ± 0.0034	0.0018 ± 0.0038	0.0020 ± 0.0041	0.0034 ± 0.0053	0.042*	(I)	0.303	0.0026 ± 0.0045	0.0029 ± 0.0047	0.0024 ± 0.0045	0.0034 ± 0.0036	0.291	(I)	0.424
Dorea	0.0094 ± 0.0091	0.0103 ± 0.0084	0.0109 ± 0.0086	0.0102 ± 0.0119	0.465	(D)	0.509	0.0075 ± 0.0070	0.0082 ± 0.0077	0.0088 ± 0.0073	0.0113 ± 0.0084	0.025*	(I)	0.112
Eggerthella	0.0017 ± 0.0031	0.0015 ± 0.0022	0.0012 ± 0.0018	0.0018 ± 0.0041	0.196	(D)	0.496	0.0024 ± 0.0033	0.0025 ± 0.0045	0.0013 ± 0.0018	0.0009 ± 0.0015	0.004	(D)	0.038**
Enterococcus	0.0006 ± 0.0015	0.0017 ± 0.0126	0.0006 ± 0.0026	0.0009 ± 0.0056	0.045*	(D)	0.303	0.0014 ± 0.0075	0.0014 ± 0.0069	0.0004 ± 0.0009	0.0007 ± 0.0015	0.223	(I)	0.366
Erysipelotrichaceae_incertae_sedis	0.0056 ± 0.0157	0.0029 ± 0.0075	0.0026 ± 0.0062	0.0028 ± 0.0062	0.093	(D)	0.387	0.0048 ± 0.0118	0.0033 ± 0.0072	0.0020 ± 0.0046	0.0011 ± 0.0027	0.003**	(D)	0.038**
Escherichia/Shigella	0.0035 ± 0.0089	0.0034 ± 0.0138	0.0045 ± 0.0185	0.0073 ± 0.0231	0.489	(D)	0.419	0.0023 ± 0.0106	0.0026 ± 0.0085	0.0029 ± 0.0114	0.0126 ± 0.0254	0.396	(I)	0.469
Faecalibacterium	0.0759 ± 0.0685	0.0768 ± 0.0549	0.0650 ± 0.0475	0.0752 ± 0.0545	0.315	(D)	0.496	0.0866 ± 0.0584	0.0904 ± 0.0584	0.0809 ± 0.0502	0.1098 ± 0.0419	0.223	(I)	0.366
Flavonifractor	0.0026 ± 0.0038	0.0023 ± 0.0029	0.0021 ± 0.0024	0.0022 ± 0.0027	0.303	(D)	0.496	0.0034 ± 0.0040	0.0031 ± 0.0032	0.0022 ± 0.0031	0.0015 ± 0.0017	<0.001**	(D)	0.014**
Fusicatenibacter	0.0166 ± 0.0228	0.0264 ± 0.0232	0.0233 ± 0.0216	0.0191 ± 0.0170	0.491	(I)	0.509	0.0248 ± 0.0231	0.0263 ± 0.0268	0.0264 ± 0.0214	0.0277 ± 0.0224	0.217	(I)	0.366
Fusobacterium	0.0173 ± 0.0576	0.0079 ± 0.0229	0.0123 ± 0.0300	0.0072 ± 0.0251	0.566	(I)	0.566	0.0048 ± 0.0227	0.0014 ± 0.0062	0.0051 ± 0.0184	0.0008 ± 0.0030	0.199	(D)	0.366
Gemmiger	0.0292 ± 0.0250	0.0192 ± 0.0196	0.0177 ± 0.0213	0.0210 ± 0.0192	0.171	(D)	0.496	0.0264 ± 0.0230	0.0286 ± 0.0244	0.0267 ± 0.0214	0.0462 ± 0.0254	0.072	(I)	0.238
Holdemanella	0.0058 ± 0.0138	0.0078 ± 0.0148	0.0098 ± 0.0175	0.0053 ± 0.0105	0.397	(I)	0.508	0.0056 ± 0.0135	0.0060 ± 0.0141	0.0056 ± 0.0113	0.0141 ± 0.0133	0.101	(I)	0.258
Intestinibacter	0.0014 ± 0.0022	0.0022 ± 0.0042	0.0022 ± 0.0057	0.0014 ± 0.0025	0.252	(D)	0.496	0.0030 ± 0.0046	0.0032 ± 0.0050	0.0039 ± 0.0070	0.0045 ± 0.0057	0.325	(I)	0.453
Klebsiella	0.0003 ± 0.0008	0.0017 ± 0.0108	0.0010 ± 0.0061	0.0004 ± 0.0017	0.340	(D)	0.496	0.0009 ± 0.0054	0.0009 ± 0.0070	0.0007 ± 0.0037	0.0000 ± 0.0000	0.448	(I)	0.501
Lachnospiracea_incertae_sedis	0.0179 ± 0.0164	0.0192 ± 0.0140	0.0179 ± 0.0136	0.0187 ± 0.0127	0.465	(I)	0.509	0.0210 ± 0.0134	0.0204 ± 0.0123	0.0232 ± 0.0147	0.0218 ± 0.0076	0.216	(I)	0.366
Lactobacillus	0.0043 ± 0.0209	0.0020 ± 0.0095	0.0043 ± 0.0279	0.0024 ± 0.0098	0.391	(D)	0.508	0.0018 ± 0.0070	0.0030 ± 0.0151	0.0036 ± 0.0128	0.0016 ± 0.0028	0.111	(I)	0.258
Megamonas	0.0129 ± 0.0327	0.0128 ± 0.0374	0.0269 ± 0.0574	0.0171 ± 0.0403	0.208	(I)	0.496	0.0081 ± 0.0412	0.0053 ± 0.0230	0.0089 ± 0.0328	0.0108 ± 0.0305	0.234	(I)	0.371
Megasphaera	0.0044 ± 0.0102	0.0048 ± 0.0116	0.0049 ± 0.0105	0.0065 ± 0.0133	0.227	(I)	0.496	0.0021 ± 0.0086	0.0030 ± 0.0091	0.0025 ± 0.0063	0.0001 ± 0.0003	0.041*	(I)	0.156*
Mitsuokella	0.0004 ± 0.0014	0.0015 ± 0.0058	0.0017 ± 0.0068	0.0054 ± 0.0234	0.332	(I)	0.496	0.0006 ± 0.0029	0.0004 ± 0.0022	0.0008 ± 0.0029	0.0000 ± 0.0000	0.268	(I)	0.404
Odoribacter	0.0008 ± 0.0011	0.0012 ± 0.0017	0.0008 ± 0.0011	0.0009 ± 0.0013	0.380	(D)	0.508	0.0015 ± 0.0017	0.0015 ± 0.0022	0.0012 ± 0.0015	0.0023 ± 0.0018	0.170	(D)	0.366
Oscillibacter	0.0056 ± 0.0087	0.0035 ± 0.0038	0.0031 ± 0.0038	0.0046 ± 0.0067	0.499	(D)	0.509	0.0055 ± 0.0061	0.0072 ± 0.0109	0.0052 ± 0.0084	0.0058 ± 0.0026	0.403	(I)	0.469
Parabacteroides	0.0160 ± 0.0305	0.0098 ± 0.0102	0.0084 ± 0.0085	0.0081 ± 0.0087	0.092	(D)	0.387	0.0094 ± 0.0100	0.0103 ± 0.0153	0.0089 ± 0.0100	0.0053 ± 0.0046	0.994	(D)	0.994
Paraprevotella	0.0031 ± 0.0072	0.0024 ± 0.0054	0.0028 ± 0.0067	0.0026 ± 0.0088	0.479	(D)	0.509	0.0017 ± 0.0043	0.0015 ± 0.0048	0.0028 ± 0.0051	0.0010 ± 0.0034	0.217	(I)	0.366
Parasutterella	0.0027 ± 0.0079	0.0042 ± 0.0096	0.0040 ± 0.0082	0.0040 ± 0.0083	0.221	(I)	0.496	0.0039 ± 0.0081	0.0045 ± 0.0092	0.0064 ± 0.0119	0.0042 ± 0.0064	0.018*	(I)	0.104*
Phascolarctobacterium	0.0056 ± 0.0066	0.0065 ± 0.0075	0.0073 ± 0.0078	0.0069 ± 0.0085	0.193	(I)	0.496	0.0044 ± 0.0058	0.0041 ± 0.0064	0.0042 ± 0.0058	0.0026 ± 0.0035	0.349	(D)	0.459
Prevotella	0.0534 ± 0.0905	0.0671 ± 0.1194	0.0880 ± 0.1349	0.1131 ± 0.1416	0.003**	(I)	0.052**	0.0330 ± 0.0789	0.0365 ± 0.0810	0.0373 ± 0.0799	0.0305 ± 0.0732	0.482	(D)	0.503
Romboutsia	0.0018 ± 0.0034	0.0050 ± 0.0082	0.0035 ± 0.0062	0.0039 ± 0.0061	0.428	(I)	0.508	0.0037 ± 0.0064	0.0048 ± 0.0092	0.0057 ± 0.0106	0.0048 ± 0.0044	0.021*	(I)	0.104*
Roseburia	0.0426 ± 0.0448	0.0440 ± 0.0379	0.0357 ± 0.0319	0.0441 ± 0.0442	0.272	(D)	0.496	0.0372 ± 0.0342	0.0440 ± 0.0416	0.0443 ± 0.0356	0.0646 ± 0.0496	0.010**	(I)	0.067**
Ruminococcus	0.0267 ± 0.0393	0.0241 ± 0.0356	0.0201 ± 0.0352	0.0280 ± 0.0383	0.338	(I)	0.496	0.0332 ± 0.0449	0.0399 ± 0.0460	0.0410 ± 0.0466	0.0623 ± 0.0560	0.006**	(I)	0.055**
Senegalimassilia	0.0011 ± 0.0021	0.0011 ± 0.0026	0.0013 ± 0.0026	0.0008 ± 0.0019	0.279	(D)	0.496	0.0007 ± 0.0018	0.0011 ± 0.0035	0.0008 ± 0.0019	0.0022 ± 0.0046	0.327	(I)	0.453
Streptococcus	0.0262 ± 0.0513	0.0148 ± 0.0244	0.0157 ± 0.0292	0.0089 ± 0.0162	0.002**	(D)	0.052**	0.0180 ± 0.0341	0.0141 ± 0.0274	0.0151 ± 0.0274	0.0412 ± 0.0788	0.398	(D)	0.469
Sutterella	0.0148 ± 0.0166	0.0139 ± 0.0161	0.0163 ± 0.0176	0.0133 ± 0.0161	0.395	(I)	0.508	0.0095 ± 0.0152	0.0058 ± 0.0094	0.0068 ± 0.0113	0.0032 ± 0.0048	0.008**	(D)	0.062**
Turicibacter	0.0020 ± 0.0046	0.0022 ± 0.0060	0.0025 ± 0.0098	0.0024 ± 0.0074	0.308	(I)	0.496	0.0019 ± 0.0041	0.0026 ± 0.0072	0.0033 ± 0.0093	0.0019 ± 0.0036	0.464	(I)	0.501
Veillonella	0.0087 ± 0.0218	0.0051 ± 0.0113	0.0052 ± 0.0125	0.0031 ± 0.0079	0.006**	(D)	0.087**	0.0066 ± 0.0145	0.0054 ± 0.0146	0.0060 ± 0.0129	0.0041 ± 0.0079	0.345	(D)	0.459

P<0.05 and <0.01 are indicated by * and **, respectively.

Q<0.20 and <0.10 are indicated by * and **, respectively.

(D) indicates tendency to decrease, (I) indicates tendency to increase in relation to VFA

*1 Jonckheere test was used

*2 Q values were adjusted by false discovery rate (FDR)

Supplementary Table 3. Relation of the relative abundance of human gut microbial genera and VFA or BMI

	VFA				BMI			
	Men		Women		Men		Women	
	Estimate	P value	Estimate	P value	Estimate	P value	Estimate	P value
Genus								
Acidaminococcus	0.090	0.075	0.041	0.312	0.101	0.045*	0.060	0.139
Akkermansia	-0.084	0.097	-0.054	0.186	-0.057	0.265	-0.061	0.133
Alistipes	-0.009	0.861	-0.039	0.335	-0.012	0.814	-0.004	0.927
Alloprevotella	0.037	0.466	0.010	0.797	0.008	0.868	-0.013	0.750
Anaerostipes	-0.029	0.564	-0.098	0.015*	-0.016	0.750	-0.111	0.006**
Bacteroides	-0.046	0.365	-0.062	0.128	-0.070	0.165	-0.090	0.027*
Barnesiella	-0.001	0.989	-0.054	0.185	-0.003	0.951	-0.010	0.799
Bifidobacterium	-0.135	0.008**	-0.104	0.010**	-0.104	0.040*	-0.047	0.245
Bilophila	0.018	0.715	0.015	0.707	0.034	0.500	-0.015	0.714
Blautia	-0.154	0.002**	-0.121	0.003**	-0.087	0.086	-0.117	0.004**
Butyricicoccus	-0.035	0.493	0.010	0.814	-0.011	0.827	-0.027	0.513
Catenibacterium	0.087	0.086	0.016	0.687	0.084	0.095	0.024	0.549
Clostridium.IV	0.029	0.573	0.047	0.245	0.063	0.216	0.077	0.057
Clostridium.sensu.stricto	0.027	0.599	0.144	<0.001**	0.052	0.306	0.173	<0.001**
Clostridium.XlVa	0.028	0.587	0.018	0.663	0.009	0.866	-0.017	0.668
Clostridium.XlVb	0.090	0.076	-0.082	0.043*	0.093	0.066	-0.085	0.035*
Clostridium.XVIII	0.019	0.713	-0.056	0.168	0.013	0.793	-0.083	0.041*
Collinsella	0.057	0.259	0.015	0.718	0.015	0.761	0.021	0.612
Coprococcus	-0.025	0.618	0.085	0.035*	0.037	0.464	0.062	0.124
Dialister	0.093	0.067	0.044	0.283	0.070	0.167	0.072	0.074
Dorea	0.005	0.919	0.087	0.032*	-0.013	0.793	0.090	0.026*
Eggerthella	-0.061	0.228	-0.129	0.001**	-0.097	0.054	-0.091	0.024*
Enterococcus	-0.069	0.170	0.042	0.297	-0.106	0.036*	0.068	0.094
Erysipelotrichaceae incertae sedis	-0.055	0.274	-0.106	0.009**	-0.126	0.012*	-0.104	0.010*
Escherichia/Shigella	-0.058	0.252	0.038	0.352	-0.103	0.042*	0.040	0.324
Faecalibacterium	-0.053	0.296	0.021	0.603	0.053	0.298	-0.004	0.920
Flavonifractor	-0.028	0.577	-0.145	<0.001**	-0.080	0.113	-0.115	0.004**
Fusicatenibacter	-0.015	0.767	0.047	0.243	0.019	0.706	0.058	0.152
Fusobacterium	0.011	0.833	-0.035	0.394	-0.045	0.378	-0.062	0.126
Gemmiger	-0.073	0.148	0.059	0.148	-0.009	0.860	0.057	0.158
Holdemanella	0.047	0.358	0.078	0.054	0.110	0.030*	0.054	0.185
Intestinibacter	-0.022	0.662	0.043	0.043*	-0.031	0.541	0.096	0.018*
Klebsiella	0.012	0.807	0.023	0.575	-0.012	0.821	0.063	0.123
Lachnospiracea incertae sedis	-0.033	0.510	0.043	0.291	0.005	0.921	0.034	0.405
Lactobacillus	0.010	0.849	0.086	0.034*	0.029	0.354	0.098	0.015*
Megamonas	0.083	0.100	0.049	0.222	0.034	0.503	0.030	0.467
Megasphaera	0.036	0.480	0.114	0.005**	0.031	0.545	0.085	0.036*
Mitsuokella	0.036	0.473	0.068	0.095	0.059	0.242	0.046	0.262
Odoribacter	-0.047	0.358	-0.037	0.365	-0.024	0.632	-0.026	0.518
Oscillibacter	-0.012	0.813	0.001	0.988	0.011	0.834	0.025	0.538
Parabacteroides	-0.067	0.184	-0.046	0.258	-0.072	0.158	-0.052	0.199
Paraprevotella	0.007	0.888	0.046	0.259	0.013	0.796	0.046	0.258
Parasutterella	0.050	0.323	0.099	0.015**	0.060	0.234	0.122	0.003**
Phascolarctobacterium	0.065	0.199	-0.026	0.521	0.026	0.615	-0.017	0.684
Prevotella	0.007	0.888	0.010	0.797	0.168	<0.001**	0.005	0.904
Romboutsia	0.014	0.781	0.109	0.007**	0.066	0.195	0.154	<0.001**
Roseburia	-0.024	0.638	0.104	0.010**	0.011	0.828	0.112	0.006**
Ruminococcus	0.014	0.778	0.091	0.025*	0.053	0.297	0.097	0.017*
Senegalimassilia	-0.016	0.755	0.050	0.217	0.058	0.257	0.047	0.245
Streptococcus	-0.002	0.960	-0.119	0.019**	-0.144	0.004**	0.007	0.871
Sutterella	0.007	0.897	-0.119	0.003**	0.077	0.130	-0.106	0.009**
Turicibacter	0.011	0.834	0.033	0.418	0.030	0.549	0.098	0.016*
Veillonella	-0.122	0.016*	-0.003	0.945	-0.147	0.004**	0.001	0.973

Spearman's correlation coefficient was used.

Supplementary Table 4. Multiple regression analysis of association among Blautia and related factors

MEN

	Estimate	P value[*1]
**VFA**	-1.09E-04	0.032*
**Age**	-2.07E-02	0.175

WOMEN

	Estimate	P value[*1]
**VFA**	-1.39E-04	0.015*
**Age**	8.55E-05	0.528
**Smoking habit**	6.08E-04	0.070
**Sleeptime**	3.20E-03	0.074
**Walk speed**	-4.28E-03	0.061

***P<0.05**

***1 Step-wise method based on AIC criterion was used**

Supplementary Table 5. Characteristics of the subjects in the confirmation group stratified by sex and VFA

Characteristics	Men	Women	P value
**Visceral fat area (cm^2)*1**	95.4 ± 47.6	63.6 ± 34.4	<0.001**
**Number**	126	210	
**Age (yr)[*1]**	50.4 ± 17.5	51.4 ± 17.6	0.623
**Height (cm)[*1]**	168.7 ± 6.8	156.0 ± 6.7	<0.001**
**Body weight (kg)[*1]**	66.6 ± 10.5	54.8 ± 9.9	<0.001**
**Body mass index (kg/m^2)[*1]**	23.3 ± 3.0	22.5 ± 3.7	0.008**
**Waist circumference (cm)[*1]**	81.9 ± 8.9	74.6 ± 9.8	<0.001**
**Serum Glucose (mg/dL)[*1]**	5.15 ± 0.91	5.13 ± 1.73	0.030*
**HbA1c (%)[*1]**	5.8 ± 0.5	5.9 ± 0.9	0.580
**SBP (mmHg)[*1]**	127.0 ± 16.0	122.7 ± 20.5	0.012
**DBP (mmHg)[*1]**	78.2 ± 11.8	72.7 ± 11.8	<0.001**
**Triglyceride (mg/dL)[*1]**	1.3 ± 0.8	1.0 ± 0.8	<0.001**
**Total cholesterol (mg/dL)[*1]**	5.0 ± 0.9	5.3 ± 1.1	0.054
**HDL cholesterol (mg/dL)[*1]**	1.5 ± 0.4	1.7 ± 0.4	<0.001**
**LDL cholesterol (mg/dL)[*1]**	2.9 ± 0.7	3.0 ± 0.9	0.478
**Smoking habits (stick/d)[*1]**	10.9 ± 11.2	2.2 ± 4.9	<0.001**
**Sleep time (h/d)[*1]**	7.1 ± 1.3	6.9 ± 1.2	0.138
**Walk speed (sec/10m)[*1]**	3.5 ± 1.0	3.9 ± 1.0	<0.001**
**Habitual medicine use (% of Yes)[*2]**	28.5%	29.0%	0.805
**Hypertension[*2]**	25.4%	24.3%	0.922
**Hyperlipidaemia[*2]**	11.1%	8.5%	0.565
**Diabetes[*2]**	7.1%	5.2%	0.634
**Rheumatism[*2]**	0.0%	0.5%	1.000
**Total energy intake (kcal/d)[*1]**	9004.1 ± 2427.5	7027.9 ± 2375.1	<0.001**
**Carbohydrate intake[*1]**	0.59 ± 0.08	0.56 ± 0.07	0.643
**Fat intake[*1]**	0.26 ± 0.06	0.28 ± 0.06	<0.001**
**Protein intake[*1]**	0.15 ± 0.03	0.16 ± 0.03	<0.001**
**Alcohol consumption (g/d)[*1]**	23.6 ± 27.7	4.5 ± 10.2	<0.001**
**Total dietary fibre intake (g/d)[*1]**	10.9 ± 4.8	10.4 ± 4.8	0.261
**Diversity of intestinal microbiota[*1]**	9.7 ± 3.3	10.1 ± 2.2	<0.001**

P<0.05 and <0.01 are indicated by * and **, respectively.

***1Wilcoxon rank-sum test was used**

***2 test for equality of proportions was used**
